# Complications With Time-to-Ambulation Following Skin Grafting for Burn Patients: A Meta-Analysis and Systematic Review

**DOI:** 10.7759/cureus.17214

**Published:** 2021-08-16

**Authors:** Tomer Lagziel, Margarita Ramos, Kevin M Klifto, Stella M Seal, Charles S Hultman, Mohammed Asif

**Affiliations:** 1 Department of Plastic and Reconstructive Surgery, Johns Hopkins University School of Medicine, Baltimore, USA; 2 Sackler Faculty of Medicine, Tel Aviv University, Tel Aviv, ISR; 3 Department of Plastic and Reconstructive Surgery, University of Pennsylvania, Philadelphia, USA; 4 Welch Medical Library, Johns Hopkins University School of Medicine, Baltimore, USA

**Keywords:** burns, enhanced recovery, ambulation, grafting, plastic and reconstructive surgery

## Abstract

Accurate models are fundamental tools for risk-stratification, therapy guidance, resource-allocation, and comparative-effectiveness research. Enhanced recovery after surgery (ERAS) protocols increase early post-operative recovery rates in surgical patients. The uniqueness of burn injuries and their post-operative care requires developing a specialized protocol, enhanced recovery after burn surgery (ERABS). To develop such a protocol, we need to examine post-operative practices, like time-to-ambulation, and their effect on post-operative complications. We evaluated evidence supporting complications such as graft loss, thrombolytic events, and pain, relating to the timing of post-surgical ambulation. A literature search on early-ambulation and skin-grafting was performed by two independent researchers. No time limit was set for publication dates. Relevant studies relating to ambulation of adult burn patients (>18 years of age) and their post-surgical outcomes were captured using search terms. Of the 888 studies retrieved from the query, 11 were used for review and meta-analysis. Our review revealed minimal evidence exists relating to thromboembolic events and time-to-ambulation in post-operative burn patients. The evidence that does exist found no significant difference in the number of events between early- and late-ambulation groups. Increased pain during rest and ambulation was shown in patients with delayed ambulation after five or more days. One study found an increased infection rate in late-ambulatory patients. The primary conclusion from this review is that further studies must be performed examining the correlation of thromboembolic events and infection rates with post-operative time-to-ambulation. Based on current literature, early ambulation should be included as part of a future model of ERABS.

## Introduction and background

Accurate models are a fundamental prognostic tool for risk stratification, therapy guidance, resource allocation, and comparative effectiveness research. The Enhanced Recovery After Surgery (ERAS) protocol is a program developed by European academic surgeons to increase early post-operative recovery rates in surgical patients [1]. ERAS protocols are multimodal management tools that take into consideration nutrition, mobilization, the timing of tube removal (e.g., urinary catheter, nasogastric tube), and the use of analgesics [2]. In the various surgical fields, unique ERAS protocols have been developed to improve the quality of specific surgeries. Overall, these protocols have been effective in reducing post-operative morbidities [3]. Burn injuries are unique and require different specialized management in comparison to other traumatic wounds [4]. Due to the unique nature of burn injuries and post-operative care, we propose that there is a need to develop a protocol unique to burn surgery, enhanced recovery after burn surgery (ERABS). In order to develop such a protocol, we need to examine multiple post-operative practices of care like time-to-ambulation and its effect on post-operative complications. Patients with lower-extremity injuries have a high risk of deep-vein-thromboses (DVTs) due to increased periods of stasis. We hypothesize that, as part of the ERABS protocol, early ambulation for lower-extremity burn patients would help reduce post-operative complications.

## Review

Materials and methods

**Figure 1:**
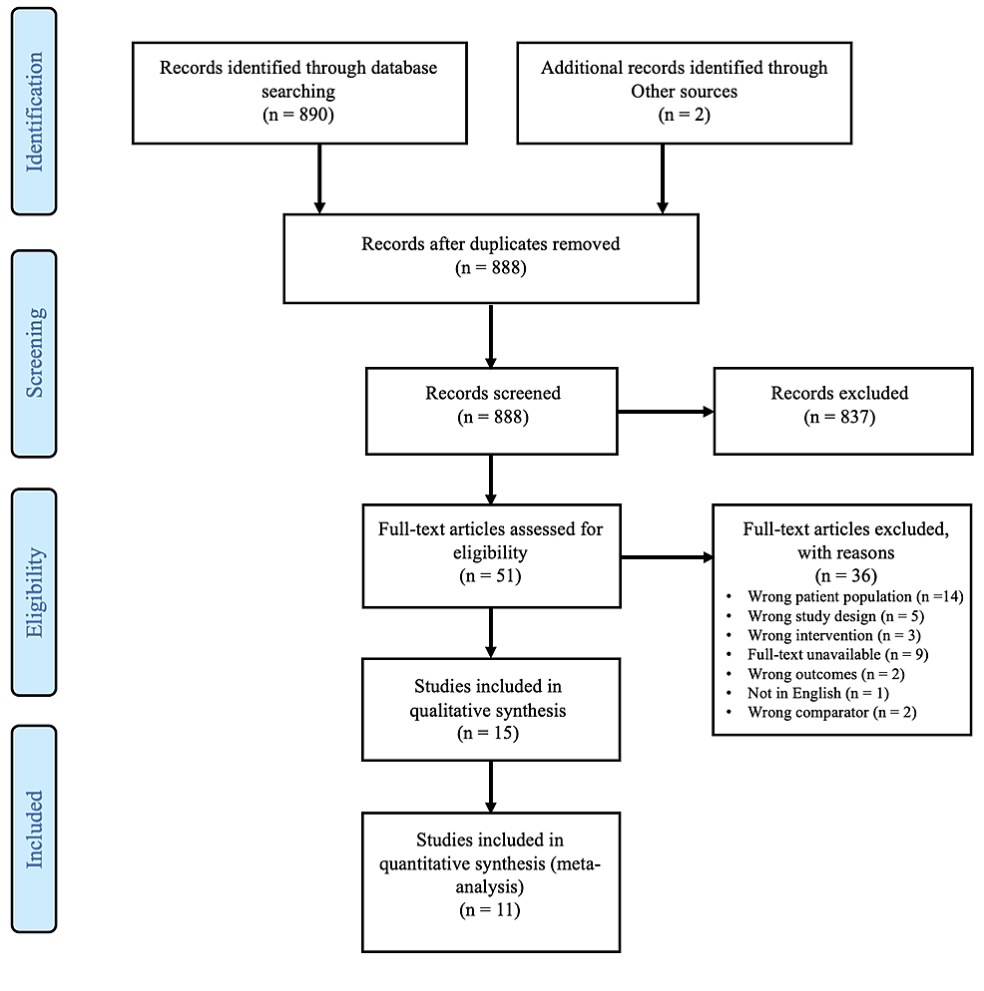
PRISMA flow chart describing the results of the final article selection and screening process. PRISMA: Preferred Reporting Items for Systematic Reviews and Meta-Analysis

Preferred Reporting Items for Systematic Reviews and Meta-Analysis (PRISMA) guidelines were followed throughout the literature search process to structure the framework for the review (Figure 1) [5]. We did not register our protocol prior to the publication of this review. 

Inclusion and Exclusion Criteria

We employed the participants, interventions, comparisons, outcomes, and study design (PICOS) strategy for study inclusion during the selection process. Participants were age over 18 years, treated as an inpatient, suffered a burn-related injury (thermal, scald, contact, electrical, chemical) of any percent total body surface area (%TBSA) or similar skin wound, to a lower extremity and required surgical intervention and grafting. Interventions were any kind of skin graft intended for the direct care of the injuries. Comparisons were of the outcomes and complications between the patients with early versus late ambulation after grafting; considering the different surgical procedures, the type and size of burn injury. Outcomes measured were %TBSA burned, wound location, depth of wound (superficial, superficial partial thickness, deep partial thickness, full thickness), mortality, overall infection, local/wound infections, line infection, pneumonia, osteomyelitis, urinary tract infection, bacteremia, sepsis, decubitus ulcer, renal failure, burn-related operations, vacuum-assisted closure (VAC), tangential excision and split-thickness skin graft, wound closure, graft loss/failure, hemodynamic complications, DVTs, respiratory complications, length of hospital stay, need for reconstruction, time to reconstruction, graft/flap loss/failure, time to management of burn, time to discharge, time to wound closure/coverage, hospital length of stay (LOS), and time period of wound closure/coverage. Study designs considered were randomized controlled trials (RCTs), non-randomized controlled trials, retrospective studies, observational studies, prospective studies, case-control studies, cohort studies, and surveys. Due to low literature availability, we decided against a predetermined length of participant follow-up or specific years of publication. Studies were excluded if they were not in English, were review articles without new academic contribution, non-reviewed peer literature, case reports, editorial articles, unavailable full-text, patient death occurred prior to hospital admission, studies observed non-extremity burns, surgical interventions were not performed.

Search of Literature

Our hospital medical library informationist (SMS), in coordination with the medical staff, conducted the preliminary literature search by means of four databases (Medline via PubMed, Embase, Cochrane, and Web of Science) with no starting limit to July 15, 2020. A list of references for relevant articles was compiled and hand-searched to identify additional applicable studies. All references were imported into Covidence (Veritas Health Innovation Ltd, Melbourne, Australia) reference management software for proper data extraction and removal of duplicate articles.

Extraction of Data

A systematic and independent title/abstract screening was carried out by two reviewers (TL and MR) and a full-text review was followed to guarantee quality and accuracy during the process. 

Any differences in an opinion involving inclusion or exclusion of a particular study were to be discussed and resolved. Any further conflict or disagreement still ongoing after the discussion would be resolved by a third reviewer (CSH). The following data points were extracted qualitatively and quantitatively: authors, year of publication, type of study, sample size, male and female distributions, age, split-thickness graft, full-thickness graft, graft loss/failure, %TBSA burned, depth of burn (superficial, superficial partial thickness, deep partial thickness, full thickness), mortality, overall infection, local/wound infections, burn-related operations, wound closure, graft loss/failure, hemodynamic complications, length of hospital stay, need for reconstruction, time to reconstruction, graft/flap loss/failure, time to management of burn, time to wound closure/coverage, hospital LOS, time period of wound closure/coverage. To avoid any duplicate reporting of results, one data collection form was used in cases where one study provided multiple reports.

Quality Assessment

The methodological index for non-randomized studies (MINORS) scale was used to separately examine the risk of bias for each study on an outcome by our two independent reviewers (TL and MR) [6]. Any further disagreement after the discussion was resolved by a third reviewer (MA). The Cochrane risk of bias tool was subsequently used to assess all of the studies across the meta-analysis. The lack of prospective studies and small sample sizes limited the assessment of risk-of-bias. In addition, the risk-of-bias was also limited due to lack of adequate reporting regarding collection methods and sample grouping. The Cochrane risk of bias tool consists of two parts, both of which were used. Firstly, it categorized risk in terms of low, high, or unclear risk. Secondly, it assessed the grading of recommendations, assessment, development, and evaluation (GRADE) quality of evidence and categorized the evidence in terms of high, moderate, low, and very low quality of evidence. All studies available meeting criteria were used in data synthesis.

Analysis Discrepancies

When time-to-ambulation was reported, there was no clear definition across all studies. In addition, some studies only defined early or late ambulation with reported for only that defined group. We extracted the data from the studies in the manner that the authors reported it. We synthesized the time-to-ambulation using a generalized definition for the two groups to be able to adequately observe all patient groups. We defined infections as a total of any and all infections reported in a given study. Pain level is another data variable that is reported differently across the literature. In order to avoid situations that would limit our results, we widened our search scope to include any studies that discussed pain levels, even qualitatively. We reviewed that data as it was presented by the authors that collected the data.

Statistical Analysis and Synthesis of Data

We compiled a table summarizing the findings which included the number of participants in the study, the outcomes presented, and the quality of evidence. In cases where studies only provided mean values, we used descriptive statistics to compare their outcomes. We performed our meta-analysis of the data using RevMan software, Version 5.3 (London, England: The Cochrane Collaboration), with a free license. We used the random-effects model, in our analysis, to account for any discrepancies in measurements, demographic characteristics, and study dates [7,8]. For dichotomous outcomes and complications, we used RRs with 95% confidence intervals (CIs). In situations with continuous outcomes and complications, we use standard mean differences (SMD) with 95% CI. All the outcomes in this meta-analysis were two-tailed, with a significance level setting of 0.05. We did not perform any additional analyses for this systematic review and meta-analysis.

Results

Characteristics and Study Selection

Our initial search yielded 890 citations. There were two duplicates present in the results which were removed yielding a total of 888 remaining citations. Initial title and abstract screening resulted in 837 irrelevant citations, leaving 51 articles eligible for full-text review. The full-text review yielded 14 studies eligible for extraction and inclusion in the systematic review (Table 1). 

**Table 1:** Summary of studies included in systematic review and meta-analysis LE: lower extremity; LOS: length-of-stay; %TBSA: percent total body surface area; ICU: intensive care unit; RCT: randomized controlled trial

Author	Year	Design	No. of patients	Group characteristics	Outcomes and complications
Golden et al. [9]	1977	Interventional	10	LE burn late ambulation split-thickness skin graft	Thromboembolic events
Harnar et al. [10]	1982	Survey case series	20	LE burn early ambulation delayed ambulation late ambulation split-thickness skin graft	Hospital LOS thromboembolic events post-operative complications
Grube et al. [11]	1987	Retrospective cohort	10	LE burn early ambulation late ambulation split-thickness skin graft	Hospital LOS graft take
Burnsworth et al. [12]	1992	Retrospective cohort	58	LE burn %TBSA early ambulation late ambulation	Hospital LOS graft take
Grube et al. [13]	1992	Retrospective cohort	100	LE burn %TBSA sheet graft	Time-to-discharge graft take post-operative complications
Wood and Lees [14]	1994	Prospective RCT	75	LE burn early ambulation late ambulation split-thickness skin graft	Graft take
Holavanahalli et al. [15]	2011	Survey	159	LE burn early ambulation late ambulation	Trend of practice
Luczak et al. [16]	2012	Retrospective cohort	48	LE burn %TBSA split-thickness skin graft early ambulation late ambulation	Graft loss hospital LOS ICU LOS thromboembolic events
Lorello et al. [17]	2014	Prospective RCT	31	LE burn %TBSA split-thickness skin graft early ambulation late ambulation	Graft loss length of stay minutes-to-ambulation
Franczyk et al. [18]	2015	Retrospective cohort	154	LE burn split-thickness skin graft early ambulation with subatmospheric pressure wound therapy	Hospital LOS graft take
Gawaziuk et al. [19]	2018	Retrospective cohort	42	LE burn %TBSA split-thickness skin graft early ambulation with a compressive dressing	Time-to-discharge graft take hospital LOS ICU LOS

Of the 14 studies, only 11 presented new information as three were meta-analyses that presented information previously covered by the other studies. We did not include or obtain any unpublished, yet relevant studies. The 11 studies that were reviewed had a total of 843 patients with lower-extremity wounds that required skin grafts. These studies were published between the years 1977 and 2018 [9-19].

The most reported outcomes were % graft take (seven of 11 studies) followed by post-operative hospital stay (six of 11 studies). We found the most reported complication to be graft loss (four of 11 studies) followed by infection (three of 11) and thromboembolic events (three of 11). Of the 51 potential outcomes and complications we searched, 13 had manageable results (Table 2). Of these studies, four compared %TBSA with a mean of 8% and a range of 0.1-10% [12,13,17,19].

**Table 2:** Combined individual study results SD: standard deviation; %TBSA: percent total body surface area; LOS: length-of-stay; ICU: intensive care unit

Author	Group	Ambulation definition (mean days +/-SD)	Sample size (n)	%TBSA (mean +/-SD)	Hospital LOS (mean +/-SD)	Post-operative LOS (mean +/-SD)	ICU LOS (mean +/-SD)	Thrombo-embolic events (n)	Wound infection (n)	Graft loss (n)	Graft take (%)	Donor site pain level at rest (mean/10 +/-SD)	Graft site pain level at rest (mean/10 +/-SD)	Wound healing time (mean days +/-SD)
Golden et al. [9]	Early ambulation	2	10	-	-	-	-	0	-	-	-	-	-	-
Harnar et al. (case series) [10]	Early ambulation	1	20	-	4 +/-3.75	-	-	0	1	1	90-100% (n=16) >80% (n =2)	-	-	-
Grube et al. [11]	Early ambulation Late ambulation	2.2 +/-1.6 8.0 +/-1.1	5 5	-	-	-	-	-	0 1	-	90-100% 90-100%	-	-	-
Burnsworth et al. [12]	Early ambulation Late ambulation	1.7 +/-1.5 7.2 +/-3.1	58 -	5.2 -	10.2 +/-6.3 12.6 +/-4.4	6.9 +/-4.4 9.4 +/-3.6	-	-	-	-	96.4% -	-	-	-
Grube et al. [13]	Early ambulation	1.0 +/1.0	100	3.7 +/-4.4	-	3 (n=64)	-	-	-	3	>85%	-	-	-
Wood Lees [14]	Early ambulation Late ambulation	1 10	36 39	-	-	4.8 +/-6.6 4.2 +/-3.4	-	0 0	-	-	82.4% 90.0%	-	-	26.5 +/-12.7 26.1 +/-17.8
Holavanahalli et al. [15]	Early ambulation Late ambulation	<5 5<	75% 25%	-	-	-	-	-	-	-	-	-	-	-
Luczak et al. [16]	Early ambulation Late ambulation	1.84 5.21	25 25	-	7.72 13.57	3.92 7.96	-	-	0 2	5 2	88% 92%	-	-	-
Lorello et al. [17]	Early ambulation Late ambulation	1 5	17 14	3.59 +/-2.06 4.07 +/-4.18	16.2 +/-3.4 17.7 +/-9.2	-	-	-	-	2 3	-	2.7 +/-2.7 4.7 +/-3.1	1.3 +/-1.8 3.5 +/-3.14	-
Franczyk et al. [18]	Early ambulation	2	154	-	-	<6 (90%)	-	-	-	-	-	-	-	-
Gawaziuk et al. [19]	Early ambulation	0.3 +/-0.6	42	5.3 +/-2	5.7 +/-8.0	6.3 +/-5.2	13.7 +/-9.1	-	-	-	98.90%	-	-	-

Three studies were found to compare total hospital stay for the early ambulation and late-to-ambulation groups. These studies defined early time-to-ambulation as an average of 2.3 days and a late time-to-ambulation as 6.4 days. These results showed an average hospital LOS of 8.2 days for the early ambulation group and 12.93 days for the late ambulation group [12,16,17]. 

Only three studies examined the presence of thromboembolic events and time-to-ambulation in post-operative patients and the results they presented were zero events for any time-to-ambulation group [9,10,14].

One study presented significant results for pain levels where patients with delayed ambulation, after five or more days, were found to have increased pain levels at rest (P = 0.02) and when ambulating (P = 0.08) [17]. Another study found increased infection rate in late-ambulatory (after five days post-operative) patients (P = 0.22) [16].

Risk of Bias and Results for Individual Studies

In Table 1, we have summarized the years, study designs, numbers of patients, group characteristics, outcomes, and complications measured by every study from the 11 articles included in our study. In Table 2, we have summarized the specifics for the important outcomes and complications we examined in each study. Unfortunately, there was little availability of RCTs in our literature search which meant that, of the seven risk of bias domains, we did not assess incomplete outcome data, selective outcome reports, blinding of participants and personnel, allocation concealment, blinding of outcome assessment, and sequence generation. Given the type and quality of the studies we found in our search, we anticipated risk of bias. None of the studies we reviewed in our meta-analysis reported patient follow-up or received perfect scores. In order to account for these various discrepancies, we decided that the optimal approach would be to utilize the “other bias” functionality in the Cochrane risk of bias tool. As seen in Figure 1, the studies received a result of “unclear bias.” The Cochrane risk of bias tool has a second functionality which gives a GRADE for the evidence of results in the studies and all received “very low” quality at the outcome level (Table 2).

Risk of Bias and Synthesis of Results Across Studies

**Figure 2:**
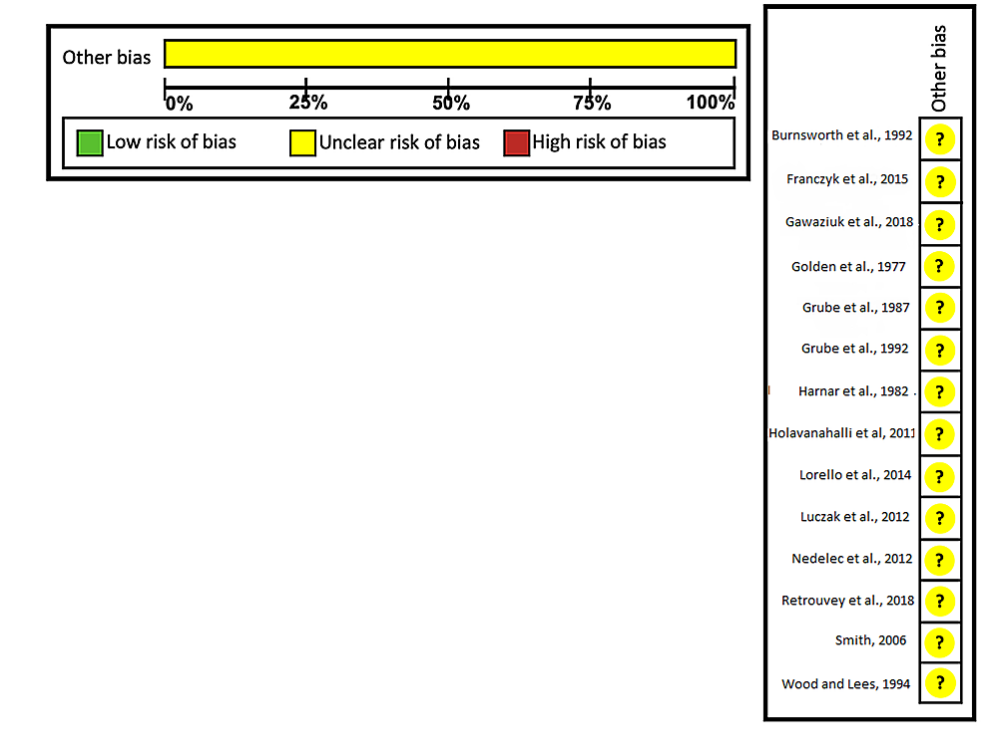
Risk of bias summary and graph with review of authors’ judgments regarding the risk of bias for each included study. Source: Refs. [9-22]

The 11 studies we decided to include in our meta-analysis were published from 1977 through 2018. They involved a total of 326 hospitalized patients with predominantly burn-related, lower extremity wounds requiring surgical skin grafting. One of the studies was a survey and did not directly collect patient data. Of the 550 patients, 409 were early post-operative mobilization patients. A delayed or late ambulation was considered standard treatment. The study designs of the 11 chosen studies were six retrospective, two prospective, one interventional, one case-series, and one survey published in English. Of the 51 potential complications and outcomes we assessed, we were able to identify 13 in the literature and we deemed them suitable for meta-analysis (Figure 2) [9-22].

Combined Study Outcomes

**Figure 3:**
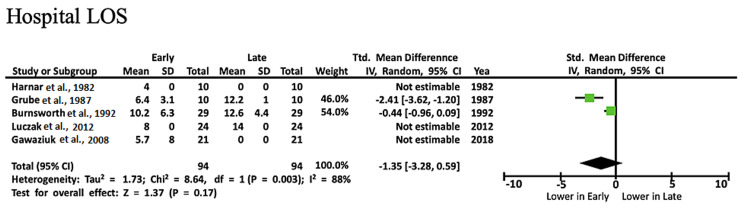
Forest plot with comparisons of complications and outcomes for hospital LOS Source: Refs. [10-12,16,19] LOS: length of stay

Hospital LOS: Five studies examined hospital LOS. Only two studies provided statistically significant results that were usable in the meta-analysis. The mean hospital LOS ranged from four to 10.2 for 94 early ambulation patients and from 12.2 to 14.4 for 94 late ambulation patients. The mean hospital LOS was longer in the late-ambulation group (SMD: -1.35, CI: -3.28, 0.59, I2= 88%, P = 0.003) (Figure 3) [10-12,16,19]. 

**Figure 4:**
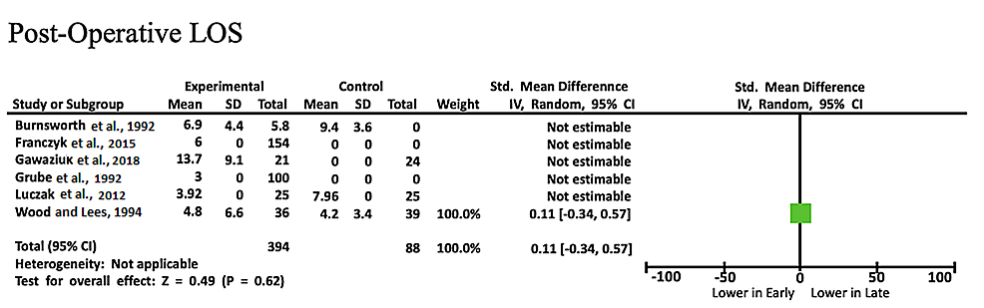
Forest plot with comparisons of complications and outcomes for post-operative LOS Source: Refs. [12-14,16,18,19] LOS: length of stay

Post-operative LOS: Six studies evaluated post-operative LOS. Only one study provided statistically significant results that were usable in the meta-analysis. The mean post-operative LOS ranged from three to 13.7 for 394 early ambulation patients and from 4.2 to 7.96 for 88 late ambulation patients. The mean post-operative LOS was longer in the early-ambulation group (SMD: 0.11, CI: -0.34, 0.57, Z = 0.49, P = 0.62) though this was not statistically significant as most studies did not have enough power or did not relay information for both study groups (Figure 4) [12-14,16,18,19].

**Figure 5:**
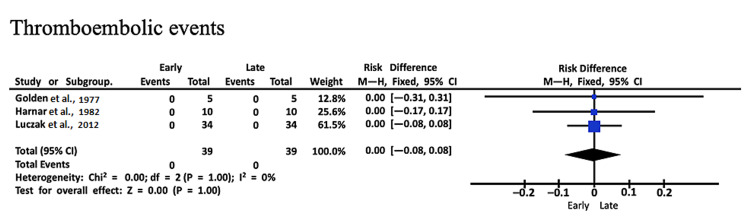
Forest plot with comparisons of complications and outcomes for thromboembolic events Source: Refs. [9,10,16]

Thromboembolic events: Three studies evaluated thromboembolic events. All studies reported zero events for both study groups. For 39 early ambulation patients, a total of zero events were reported as well as zero total events for 39 late ambulation patients. The mean events for both groups was zero (SMD: 0.00, CI: -0.08, 0.08, I2 = 0%, P = 1.00) (Figure 5) [9,10,16].

Wound infections: Only three studies reported comparing wound infections. However, the sample sizes in the studies were not sufficient for meta-analysis. However, as observed in the combined table, more wound infections were found in the late-ambulation group (Table 2).

Pain levels: One study examined donor and graft site pain levels in both early and late ambulation patients. The results can be viewed in the combined table (Table 2). In this study, the authors used that standardized Visual Analogue Scale (VAS) score to assess patient pain levels. Since this was the only study found to examine this data point, it could not be compared to other studies and a meta-analysis could not be performed. However, this study found lower pain levels in the early ambulation group.

Discussion

Time-to-ambulation has been a greatly discussed topic in current literature. In the past, there was a systematic review that solely investigated graft loss in lower-extremity wounds without examining other complications. In addition, our review and meta-analysis aimed to dive deeper into burn-related wounds and included a variety of different studies. More studies are being done to understand the health benefits of early post-operative mobilization [23]. In patients with lower extremity wounds that require split-thickness skin grafts, most post-operative management protocols prefer early mobilization but, in practice, this is not always the case [17,20]. Early post-operative ambulation involves decreasing hospital stay which could result in decreased costs for the patient and the hospital. This would also mean that much of the post-operative patient management would be translated from inpatient to outpatient. Given the complexity of lower-extremity burn wounds, wound care and hygiene are crucial in the prevention of complications like graft loss or infection. Proper wound management is essential to even allow for early ambulation to take place. When we analyzed the studies that reviewed the topic of LOS, we found that there is also a discrepancy in reporting the patients’ pre-injury status as well as injury severity. In the two studies that were eligible for meta-analysis of for hospital LOS, one article reported specific mechanism of injury, %TBSA, and localization within the lower extremity whereas the other article only focused on molten injuries and did not discuss specific wound localization or %TBSA. Given this variability and significance of the specific result, we chose to analyze this data heterogeneously. We did not find articles that objectively analyzed pre-injury ambulation status which could also be a contributing factor in expediting post-injury ambulation and recovery. Generally speaking, increased stasis times are associated with an increased risk for thromboembolic events. In addition, a lengthened hospital stay is associated with an increased risk for complications like wound infection or hospital-acquired systemic morbidities. Unfortunately, the studies currently available examining different times to ambulation are extremely limited in their power. Many studies only examine one experimental group and report findings without comparison. Other studies compare their own findings from an experimental group to literature findings or to survey data without providing specific patient characteristics on the data, result in loss of statistical significance of the data. Another issue resulting in decreased data reliability was that the studies ranged from 1977 to 2018, covering a large time period where inpatient management has evolved, and new grafting techniques have been developed and implemented. The patient populations examined also varied from study to study, resulting in possible bias and decreased reliability. This large amount of limitations could rationalize the unclear degree of heterogeneity and unclear bias observed. While DVTs and time-to-ambulation in burn-related lower extremity injuries have been extensively researched, studies show that, in general, early mobilization is favored in reducing the risk for DVTs [24]. The importance of carrying out this systematic review and meta-analysis is made evident by these very clear limitations. Even though we came across some associations, the lack of rigorous methodologies, the poor quality of study, and various confounding variables negatively affect many of the observed outcomes, and any conclusions drawn should be heavily considered. We do positively highlight the use of standardized scales for patient assessment, as seen with pain levels using the VAS score, as this allows objective literature review and more fluent comparison between studies. While different standardized scales for pain assessments exist, most are considered reliable [25]. Any future studies that aim to assess pain levels in post-operative conditions should use a standardized pain scale. Furthermore, without confirming our findings through additional, powerful prospective studies we cannot entirely attribute any observed outcomes or associations to the effects of time-to-ambulation.

## Conclusions

A significant limitation that we have found in the existing literature is that there are no standardized definitions exist for early or late post-operative ambulation. We have recognized potential future fields of research, through performing this review and analysis, that will be able to provide an abundance of data to the literature for medical professionals and to patients alike. Furthermore, using PRISMA and Cochrane guidelines, we were able to compare differences in complications and outcomes between different studies using the highest levels of evidence available. The primary conclusion from this review is that further, extensive, prospective randomized control studies need to be performed examining the correlation of thromboembolic events and infection rates with post-operative time-to-ambulation. Based on current literature, early ambulation should be included as part of a future model of ERABS because a shorter length of stay lowers the risk for hospital-acquired infections. Also, reduced associated pain levels could lead to decreased risk for opioid dependence.
